# FibroChip, a Functional DNA Microarray to Monitor Cellulolytic and Hemicellulolytic Activities of Rumen Microbiota

**DOI:** 10.3389/fmicb.2018.00215

**Published:** 2018-02-13

**Authors:** Sophie Comtet-Marre, Frédérique Chaucheyras-Durand, Ourdia Bouzid, Pascale Mosoni, Ali R. Bayat, Pierre Peyret, Evelyne Forano

**Affiliations:** ^1^UMR 454 MEDIS, INRA, Université Clermont Auvergne, Clermont-Ferrand, France; ^2^R&D Animal Nutrition, Lallemand, Blagnac, France; ^3^Milk Production Solutions, Green Technology, Natural Resources Institute Finland (Luke), Helsinki, Finland

**Keywords:** rumen, cellulolysis, hemicellulolysis, glycoside hydrolases, carbohydrate esterases, functional DNA microarray, metatranscriptomics

## Abstract

Ruminants fulfill their energy needs for growth primarily through microbial breakdown of plant biomass in the rumen. Several biotic and abiotic factors influence the efficiency of fiber degradation, which can ultimately impact animal productivity and health. To provide more insight into mechanisms involved in the modulation of fibrolytic activity, a functional DNA microarray targeting genes encoding key enzymes involved in cellulose and hemicellulose degradation by rumen microbiota was designed. Eight carbohydrate-active enzyme (CAZyme) families (GH5, GH9, GH10, GH11, GH43, GH48, CE1, and CE6) were selected which represented 392 genes from bacteria, protozoa, and fungi. The DNA microarray, designated as FibroChip, was validated using targets of increasing complexity and demonstrated sensitivity and specificity. In addition, FibroChip was evaluated for its explorative and semi-quantitative potential. Differential expression of CAZyme genes was evidenced in the rumen bacterium *Fibrobacter succinogenes* S85 grown on wheat straw or cellobiose. FibroChip was used to identify the expressed CAZyme genes from the targeted families in the rumen of a cow fed a mixed diet based on grass silage. Among expressed genes, those encoding GH43, GH5, and GH10 families were the most represented. Most of the *F. succinogenes* genes detected by the FibroChip were also detected following RNA-seq analysis of RNA transcripts obtained from the rumen fluid sample. Use of the FibroChip also indicated that transcripts of fiber degrading enzymes derived from eukaryotes (protozoa and anaerobic fungi) represented a significant proportion of the total microbial mRNA pool. FibroChip represents a reliable and high-throughput tool that enables researchers to monitor active members of fiber degradation in the rumen.

## Introduction

Ruminants are among the most efficient herbivorous animals to convert plant biomass into edible products, principally due to a symbiotic relationship with microorganisms inhabiting the rumen. The rumen microbiota is composed of a very diverse and complex population of bacteria, archaea, protozoa, and fungi. This microbial community has a remarkable ability to degrade and ferment complex plant polymers and thereby provides its host with protein and volatile fatty acids which represent an important nitrogen and energy source for the animal. The efficiency of the plant biomass degradation depends on the activity of the microbial enzymes produced by the rumen microorganisms. These enzymes include glycoside hydrolases (GH) active against the main plant structural polysaccharides (cellulose and hemicelluloses) and carbohydrate esterases (CE) which cleave polysaccharide substituents (Flint et al., 2012). Most of these enzymes are organized in a modular structure including one or several catalytic domain(s) bearing the hydrolytic activity and accessory domains involved in various functions such as substrate binding [carbohydrate binding module (CBM)] or cellulosome assembly (Gilbert, 2010; White et al., 2014; Artzi et al., 2017). Fibrolytic activity of rumen microorganisms can be affected by diverse biotic or abiotic factors, such as chemical composition and physical structure of the plant material, use of dietary additives, or microbiota dysbiosis leading to unfavorable ruminal conditions (Chaucheyras-Durand et al., 2012).

A few cellulolytic and hemicellulolytic bacterial species are considered as major biomass degraders in the rumen (White et al., 2014). Sequencing of their genome has revealed a high number of genes which could be involved in plant fiber degradation. Also, metagenomic approaches have given an insight into the genes collectively present in the rumen and potentially implicated in this process (Brulc et al., 2009; Hess et al., 2011; Wang et al., 2013; Güllert et al., 2016; Pitta et al., 2016). However, these approaches only identify a degradation potential of the rumen microbiota and do not identify the enzymes actually produced *in vivo* or the regulation of their expression. Recently, metatranscriptomic approaches were applied to the rumen microbiota to identify the key actors in the plant fiber deconstruction (Dai et al., 2015; Shinkai et al., 2016; Comtet-Marre et al., 2017). These studies described the main carbohydrate-active enzyme (CAZyme) families involved in this process under their experimental conditions, but also underlined the lack of gene catalogs to give a complete view of the rumen microbiota at the taxonomic and functional level. At a larger scale, knowledge gaps remain on how the activity of rumen fibrolytic microorganism is modulated by various factors at the transcriptomic level.

DNA microarrays have been extensively used as very sensitive and high-throughput tools for exploring the structure of complex microbial ecosystems (Kang et al., 2013; Tottey et al., 2013) as well as investigating functional gene sets (Dugat-Bony et al., 2012a; Tu et al., 2014). However, their use has been drastically decreased since the rise of new generation sequencing technologies applied to metagenomics and metatranscriptomics. Indeed, these non-targeted approaches enable the detection of millions of genes or transcripts present in the studied ecosystem, that have been already identified or not, and that appear of interest or not. On the contrary, microarrays generally focus on a determined number of known genes and also on their genetic variants in the case of explorative microarrays (Dugat-Bony et al., 2012b). Functional gene arrays (FGA), which are microarrays containing probes for key genes involved in microbial functional processes (He et al., 2012; Abot et al., 2016), present several advantages for specific applications, including (i) they target functions or metabolic pathways of interest; (ii) they allow to monitor in parallel and at low cost a high number of samples such as those generated by studies on large number of animals or kinetics, and (iii) up to now, analysis of results has been also easier and quicker than for metatranscriptomic data.

In the present work, we developed a FGA targeting fiber-degrading activities in the rumen ecosystem, designated as FibroChip. The microarray was designed to target genes belonging to eight CAZyme families known to contain very efficient cellulases and hemicellulases (i.e., GH5, GH9, GH10, GH11, GH43, GH48, CE1, and CE6) which are present in the genome of the major rumen fibrolytic microorganisms including bacteria, fungi, and protozoa. The DNA microarray allows the detection of 392 genes and is composed of 4249 probes. We present here the validation of this FGA using targets of increasing complexity and its use to analyze the transcriptome of one of the major rumen cellulolytic bacterium *F. succinogenes.* We also applied this new tool to characterize the expression of the CAZyme gene repertoire of the rumen microbiota of a dairy cow and compared the results with a RNA-sequencing (RNA-seq) approach. The FibroChip demonstrated sensitivity and specificity as well as explorative potential.

## Materials and Methods

This work was undertaken according to the relevant biosecurity and INRA safety procedures.

### Sequence Retrieval, Probe Design, and Microarray Features

For all rumen microorganisms known to degrade cellulose, hemicelluloses, and oligosaccharides, nucleic sequences encoding the CAZyme families GH5, GH9, GH10, GH11, GH43, GH48, CE1, and CE6 were extracted from the CAZy database (**RRID**:SCR_012909^1^; Lombard et al., 2014) when available (Supplementary Table S1). Sequences from environmental anaerobic microorganisms not yet known to be part of the rumen microbiota were also included. Probes were designed specifically in the coding sequence of the catalytic domain of each gene. Consensus sequences were determined for sequences with more than 96% of identity that should not be distinguished with the microarray. In this particular case, probes were designed in conserved regions and targeted a set of genes instead of a unique gene. Sense oligonucleotide probes were designed using the HiSpOD software (**RRID**:SCR_014403^2^; Dugat-Bony et al., 2011). Parameters used were: probe length = 25-mers, Tm min = 46°C, Tm max = 64°C, and Complexity = 10. Specificity of deduced probes was tested against the EnvExBase database implemented in the HiSpOD software, and probes with more than 85% of identity and more than 15 consecutive bases (similarity stretch) with a non-targeted sequence were discarded by following the criteria of Kane et al. (2000) and He et al. (2005). Probes presenting any potential cross-hybridization with coding sequences from gut or anaerobic microorganisms were also discarded. Remaining probes were further evaluated by a BLASTN (**RRID**:SCR_001598; Camacho et al., 2008; BLAST+ 2.2.23) against rDNA sequences of the NCBI non-redundant database (release 176; **RRID**:SCR_004860) to ensure specificity of probes (*e*-value <1e-05). Depending on the length of the targeted sequence and the possibilities to determine specific probes, we selected between 2 and 20 25-mer probes per gene, distributed along the target (Supplementary Table S2). To enhance the sensitivity of the microarray while keeping specificity, we also used the GoArrays strategy (**RRID**:SCR_005785^3^; Rimour et al., 2005). To this end, selected probes were concatenated to produce 54-mer probes, constituted of two 25-mer probes linked by a spacer of 4 nt (Supplementary Table S2). Triplicate of probes of 25- and 54-mers were synthetized *in situ* on an Agilent 8 × 15K DNA microarray (Agilent Technologies, Santa Clara, CA, United States) allowing the simultaneous analysis of 16 biological samples using a two color labeling. The microarray contained also 382 Agilent internal control probes including positive controls, negative controls, and quality control probes. Probes were randomly placed on the array to avoid position bias. The 392 CAZymes whose genes are targeted by the FibroChip microarray are presented in Supplementary Table S3.

### DNA Hybridization Experiment

#### Strains and Culture Conditions

Strains *F. succinogenes* S85 (ATCC 19169), *Ruminococcus albus* 20 (ATCC 27211), *R. albus* 7 (ATCC 27210), and *Bacteroides xylanisolvens* XB1A (DSM 18836T) were grown 15 h in a complex medium containing 20% of clarified rumen fluid (Béra-Maillet et al., 2000) and 0.2% of cellobiose (Clb) (Sigma–Aldrich, Saint-Louis, MO, United States) or 0.5% of wheat straw (WS) as carbon source, at 39°C for the two first species and 37°C for the third one. The fungal strain *Piromyces* sp. M4 was isolated in our laboratory from a hay-fed rumen cannulated sheep. The monocentric strain was selected from bacterium-free culture enrichments in the complex medium of Orpin (1975) supplemented with a mixture of antibiotics and a strip of filter paper (Whatman No. 1) as previously described (Chaucheyras-Durand et al., 2010). Genus affiliation was derived from sequencing of the internal transcribed spacer region 1. All microbes were cultured under strictly anaerobic conditions (Hungate, 1950). The strain *Escherichia coli* K12 (ATCC 10798) was cultivated 15 h at 37°C in Luria-Bertani medium under shaking and aerobic conditions.

#### DNA Isolation

Cultures of bacterial strains (10 ml) were centrifuged for 20 min at 10,000 ×*g* and bacterial pellets were washed three times with 2 ml of phosphate-buffered saline (PBS). Before DNA extraction, the bacterial pellet was incubated 5 min in 200 μl of PBS and 1 μl of proteinase K (20 mg/ml; Invitrogen, Thermo Fisher Scientific, Waltham, MA, United States). Bacteria were disrupted for 3 min with ∼100 mg of 0.1 mm Zirconia Beads (BioSpec Products, Bartlesville, OK, United States) using the model MM2000 BeadBeater instrument (Retsch, Eragny-sur-Oise, France). Then, DNA extraction was performed using the EasyDNA Kit (Invitrogen, Thermo Fisher Scientific, Waltham, MA, United States) following the manufacturer instructions. Concentration and quality of isolated genomic DNA (gDNA) were checked with the spectrophotometer Nanodrop 1000 (Thermo Fisher Scientific, Waltham, MA, United States). DNA integrity was verified by 1% agarose gel electrophoresis, and the samples were stored at -20°C.

#### Cloning of Genes Encoding Fibrolytic Enzymes

Genes from *F. succinogenes* S85, *R. albus* 7, and *Piromyces* sp. M4 (Supplementary Table S4) were PCR amplified from 200 ng of gDNA using a high-fidelity DNA polymerase (Pfx50 DNA Polymerase, Invitrogen, Thermo Fisher Scientific, Waltham, MA, United States). PCR products of expected length were purified from electrophoresis gel matrix using the QIAquick Gel Extraction Kit Protocol (Qiagen, Hilden, Germany) according to the supplier instructions. Purified PCR products were cloned in the PCRII-TOPO vector (Invitrogen, Thermo Fisher Scientific, Waltham, MA, United States). *E. coli* (One Shot TOP10 Chemically Competent *E. coli*, Invitrogen, Thermo Fisher Scientific, Waltham, MA, United States) were then transformed with recombinant plasmids. Plasmids encoding *cel9B* from *R. albus* 20, *xyn10A* from *B. xylanisolvens* XB1A, and *xynB* from *Polyplastron multivesiculatum* were from previous work (Supplementary Table S4). Recombinant cells were cultivated in LB medium with ampicillin (100 μg/ml) or kanamycin (50 μg/ml). Plasmids were extracted with the QIAprep MiniPrep Kit (Qiagen) from 5 ml of a 15 h culture of *E. coli* incubated at 37°C under shaking conditions. Inserts were sequenced to ensure that cloned DNA fragments had the same sequence as the genes used for probe design. Cloned genes were then PCR amplified with the Pfx50 DNA Polymerase (Invitrogen, Thermo Fisher Scientific, Waltham, MA, United States) using primers listed in Supplementary Table S4 and PCR products were gel purified as described above.

#### Microarray Target Labeling and Hybridization

Genomic DNA from each bacterial strain (2 μg) was sheared in a 200-μl milliQ water volume by 10 s of sonication (Vibra Cell VC300, Sonics & Materials, Inc., Newtown, CT, United States). The length of sheared gDNA (range between 500 and 1500 bp) was checked by 1% agarose gel electrophoresis. Fragmented gDNA was labeled using the Bioprime Total Genomic Labeling Kit and Alexa Fluor 3 or 5 dyes (Invitrogen, Thermo Fisher Scientific, Waltham, MA, United States) following the supplier’s instructions. A degree of labeling higher than 0.7 for the Alexa Fluor 3 and 1.2 for the Alexa Fluor 5 was considered as acceptable.

Each PCR product (2 μg) was labeled independently using the ULS aRNA Labeling Kit and Cy3 or Cy5 cyanines (Kreatech Diagnostics, Amsterdam, Netherlands) following the manufacturer’s recommendations. A degree of labeling between 1.0 and 2.5% was considered as acceptable. DNA hybridization (1 μg for each DNA and 50 ng for each PCR product) was performed at 65°C during 24 h following the Agilent protocol “Oligonucleotide Array-Based CGH for gDNA Analysis”.

### RNA Hybridization Experiment

#### Strain and Culture Conditions

The strain *F. succinogenes* S85 was grown with either 0.2% of Clb (Sigma–Aldrich, Saint-Louis, MO, United States) under static conditions or 0.5% of WS under shaking conditions. The strain was cultured in triplicate on the same medium. Cultures were harvested at the end of the exponential growth after 6 and 50 h, respectively (Béra-Maillet et al., 2000; Nouaille et al., 2009).

#### Animal Experiment, Diet, and Sampling of Rumen Contents

All experimental procedures were approved by the National Ethics Committee (Hämeenlinna, Finland) in accordance with the guidelines established by the European Community Council Directive 86/609/EEC (Louhimies, 2012). The analyzed rumen sample was obtained from a rumen cannulated lactating dairy cow fed a total mixed ration based on grass silage (forage:concentrate ratio 50:50, on a dry matter basis). Details of the diet fed have been reported previously (Bayat et al., 2015). In brief, the total mixed ration contained (gram per kilogram dry matter) neutral detergent fiber (401), acid detergent fiber (228), and starch (120). Samples were taken before the morning feeding from five different sites in the rumen, composited, and mixed thoroughly to obtain a representative sample of rumen contents. For molecular analysis, rumen content was subsampled (50 g) and mixed with 100 ml of RNAlater (Thermo Fisher Scientific, Waltham, MA, United States) to prevent RNA degradation. The mixture was maintained overnight at +4°C and then stored at -80°C until nucleic acid extraction.

#### RNA Isolation and mRNA Enrichment

Total RNA from *F. succinogenes* S85 was extracted in duplicate from 10 ml of culture for both substrate conditions (Clb and WS). RNA isolation was performed using Trizol reagent (Invitrogen, Thermo Fisher Scientific, Waltham, MA, United States) with slight modifications of the manufacturer’s instructions. Cultures were centrifuged for 15 min at 12,000 ×*g* at +4°C. Then, 10 ml of Trizol was added to the pellets and the mix was vigorously vortexed. Cells were disrupted with ∼100 mg of 0.1 mm Zirconia Beads (BioSpec Products, Bartlesville, OK, United States) in a 2 ml-microtube using the FastPrep Instrument (MP Biomedicals, Irvine, CA, United States), set to 6.5 m/s, during four cycles of 30 s interspaced with 5 min of incubation on ice. Following steps were those recommended by the Trizol protocol.

Total RNA from rumen content was extracted in duplicate from 400 mg of centrifuged rumen content using Trizol as previously described (Comtet-Marre et al., 2017). The integrity of RNA was assessed with the Agilent 2100 Bioanalyzer using RNA Nano Chip (Agilent Technologies, Santa Clara, CA, United States) and quantity was assessed with the Nanodrop 1000 Spectrophotometer (Thermo Fisher Scientific, Illkirch-Graffenstaden, France). Duplicates of total RNA were pooled and the resulting RNA solutions were stored at -80°C until further processing. Total RNA from *F. succinogenes* S85 and rumen contents were submitted to mRNA enrichment using an in-house-procedure based on rRNA subtractive hybridization principle as previously described (Comtet-Marre et al., 2017).

#### Target Labeling and Hybridization

Fractions of mRNA-enriched RNA (200 ng) were amplified with the MessageAmp II-Bacteria RNA Amplification Kit (Ambion, Thermo Fisher Scientific, Waltham, MA, United States) according to the modified protocol of Dugat-Bony et al. (2012a) to incorporate aminoallyl UTP during a 14 h-*in vitro* transcription (Ambion, Thermo Fisher Scientific, Waltham, MA, United States). For each sample, 10 μg of amplified RNA (aRNA) was labeled with Cy3 or Cy5 cyanines (Pack Amersham CyDye Post-Labeling Reactive Dye, GE Healthcare, Little Chalfont, United Kingdom), following the manufacturer’s recommendations. Labeled aRNA was finally purified with NucleoSpin RNA Clean-Up Kit (Macherey-Nagel, Düren, Germany). Labeling density was acceptable when values were between 30 and 60 (one dye per every 30–60 unlabeled nucleotides). RNA hybridization (1 μg for the strain *F. succinogenes* S85 and 5 μg for rumen samples) was performed at 65°C during 17 h following the Agilent protocol “Two-Color Microarray-Based Gene Expression” with few modifications for the use of a supplementary blocking agent (Kreablock, Kreatech Diagnostics, Amsterdam, Netherlands). Briefly, RNA fragmentation was performed at 60°C during 30 min in a mix composed of 12.5 μl of Kreablock buffer (Kreatech Diagnostics, Amsterdam, Netherlands), 6.5 μl of aRNA, 5 μl of 10× Gene Expression Blocking Agent (Agilent Technologies, Santa Clara, CA, United States), and 1 μl of 25× fragmentation buffer (Agilent Technologies, Santa Clara, CA, United States). Next steps of hybridization and slide washing were performed according to Agilent protocol.

### Imaging, Data Preprocessing, and Analysis

Immediately after washing steps, microarray slides were scanned using an Agilent G2505B microarray scanner (Agilent Technologies, Santa Clara, CA, United States) with a resolution of 5 μm and the XDR mode (Extended Dynamic Range) set up at 10 and 100% PhotoMultiplier Tubes (PMT) gain. Fluorescence intensities of oligonucleotide spots were extracted from the scanned images using the Feature Extraction software (V11.0, Agilent Technologies, Santa Clara, CA, United States). Data are available under NCBI GEO (accession number GSE107550). Data were processed with dedicated scripts based on C++ and Delphi languages. For each probe, median intensity value of the three replicates was conserved and used as the probe signal value. The signal-to-noise ratio (SNR), similar to the detection threshold response and corresponding to the probe signal value divided by the local background intensity value, was calculated for each probe. Results for 25- and 54-mer probes were treated independently and for each probe type, a gene was considered as expressed when 65% of probes were positive. The SNR value of a detected transcript was then calculated by the mean of the SNR of all 25- or 54-mer probes targeting this gene. For PCR product hybridizations, gDNA hybridizations, and *F. succinogenes* RNA hybridizations, the SNR thresholds were set to 3 for 25-mer probes and 6 for 54-mer probes. For rumen RNA hybridizations, the SNR threshold was set at 2 such that a positive response was considered when the signal intensity of a probe was two times superior to the signal of the background and negative control probes (He and Zhou, 2008).

### Quantification of the Expression of Genes from *Fibrobacter succinogenes* S85

For *in vitro* cultures, *F. succinogenes* S85 total RNA (100 ng) was reverse-transcribed into cDNA using Superscript II reverse transcriptase (Invitrogen, Thermo Fisher Scientific, Waltham, MA, United States) according to the manufacturer recommendations. Three replicates were used for each substrate condition. For each RNA sample, a negative control (no addition of reverse transcriptase) was performed to assess DNA contamination.

For qPCR quantification of *F. succinogenes* S85 gene expression in the cow rumen content, mRNA enriched RNA (180 ng in duplicate) from total rumen content was reverse-transcribed in cDNA with the polymerase M-MLV RT RNase H Minus DNA (Euromedex, Souffelweyersheim, France) following manufacturer recommendations.

The different sets of cDNA were then used for qPCR quantification of the expression of *F. succinogenes* S85 genes in the *in vitro* cultures and in the rumen content. Primers used for the qPCR quantifications of *endB* (GH9 ACX73672), *endA* (GH9 ACX75948), and *xynD* (GH10 ACX75384) were those described in Béra-Maillet et al. (2009). Primers used for *axe6B* (CE6 ACX75360) amplification were Forward(5′–3′)-TGTGAGCATCCGCGCCTTCG and Reverse(5′–3′)-TTGCGCCAGTTGGCATCGGT, and primers used for *celE* (GH9 ACX 75451) cDNA quantification were Forward(5′–3′)-CAGGCTGCAGATGGTGGCGT and Reverse(5′–3′)-TTGGCCGCCTCAAGGCACTG. qPCR reactions were achieved mostly as described in Béra-Maillet et al. (2009). They were carried out in duplicate in 96-well plates using a total volume of 20 μl with 10 μl of 2× Takara SYBR Premix Ex Taq (Lonza, Bâle, Switzerland), 1 ng of cDNA, and 0.5 μM of each primer. For each cDNA quantification, standard curves were used in order to determine the absolute number of copies as described in Béra-Maillet et al. (2009). The gene copy numbers were calculated from three biological replicates and their two technical replicates.

### Isolation of a GH11 Transcript of *Piromyces communis* from Total Rumen Content

Messenger RNA enriched RNA (180 ng) from total rumen content was reverse-transcribed in cDNA as described above and using a mix of primers deduced from probes targeted the gene encoding a GH11 hemicellulase from Piromyces communis (protein accession number AAG18439). Primers were eliminated by column-based purification using the QIAquick PCR Purification Kit (Qiagen, Hilden, Germany). One-tenth of the reverse transcription reaction was used to perform a primary PCR. Then a secondary nested PCR was achieved with 1.5 μl of the first PCR reaction used as PCR template. PCR was performed using the polymerase GoTaq G2 DNA (Promega, Madison, WI, United States) following supplier recommendations. Amplification steps were: initial denaturation (94°C, 2 min), 35 cycles comprising denaturation (95°C, 30 s), hybridization (50°C, 30 s), elongation (72°C, 60 s), and final elongation (72°C, 5 min). Primers used are reported in Supplementary Table S5. Several secondary PCR reactions were realized in order to obtain enough PCR products for sequencing. PCR products were gel-purified using the QIAquick Gel extraction Kit (Qiagen, Hilden, Germany) according to the supplier instructions. Finally purified PCR products were concentrated with the MinElute PCR Purification Kit (Qiagen, Hilden, Germany) and sequenced by the MWG Company (Mulhouse, France) using primers of the secondary PCR reaction.

### RNA-Seq Data

Microarray data from rumen RNA hybridization were compared with RNA-seq data (available on Sequence Read Archive, SRP070140) obtained in a previous work (Comtet-Marre et al., 2017). Briefly, translated non-rRNA were submitted to a local version of dbCAN (**RRID**:SCR_013208) (Yin et al., 2012) to detect and annotate sequences for CAZymes (*e*-value < 1*e*-05). dbCAN positive sequences were subjected to sequence similarity searches using BLASTX (BLAST+ 2.5.0; **RRID**:SCR_001653) (Camacho et al., 2008) against the NCBI NR database protein (**RRID**:SCR_003257) (*e*-value < 1*e*-05). BLAST results (25 best hits for each read) were used for taxonomic mining of putative CAZyme transcripts using the MEGAN software version 6 (**RRID**:SCR_011942) (Huson et al., 2011). The taxonomic level was determined using the lowest common ancestor-based algorithm (LCA) implemented in the software. LCA parameters were “Top percent” set to 10, “Min support percent” 1*e*-05, and “Min score” set to 90 (Comtet-Marre et al., 2017).

## Results and Discussion

### Overall Description of FibroChip Features

The FibroChip was designed with the objective to propose a high-throughput tool providing a rapid picture of the capacity of rumen microorganisms to degrade cellulose and hemicelluloses based on a targeted metatranscriptomic approach. We chose to focus on a few number of genes by targeting main ruminal fibrolytic microorganisms and selected CAZyme families that may have a pivotal role in cellulose and hemicellulose degradation. The microarray targets the coding sequence of catalytic domains from eight CAZyme families involved in cellulose and hemicellulose degradation (i.e., GH GH5, GH9, GH10, GH11, GH43, and GH48, and CE CE1 and CE6). Taken together, these families present complementary activities needed for the complete degradation of cellulose and hemicelluloses (**Table 1**).

**Table 1:** Substrate and known activities of the CAZyme families targeted with the FibroChip.

Substrate	CAZy family	Known activities
Cellulose	GH5 (except subfamily 21)	Endo-β-1,4-glucanase/cellulase (EC 3.2.1.4); β-glucosidase (EC 3.2.1.21); β-mannosidase (EC 3.2.1.25); glucan β-1,3-glucosidase (EC 3.2.1.58); licheninase (EC 3.2.1.73); exo-β-1,4-glucanase/cellodextrinase (EC 3.2.1.74); glucan endo-1,6-β-glucosidase (EC 3.2.1.75); mannan endo-β-1,4-mannosidase (EC 3.2.1.78); cellulose β-1,4-cellobiosidase (EC 3.2.1.91); chitosanase (EC 3.2.1.132); endo-β-1,6-galactanase (EC 3.2.1.164); β-1,3-mannanase (EC 3.2.1.-); mannan transglycosylase (EC 2.4.1.-)
	GH9	Endoglucanase (EC 3.2.1.4); endo-β-1,3(4)-glucanase/lichenase–laminarinase (EC 3.2.1.6); β-glucosidase (EC 3.2.1.21); lichenase/endo-β-1,3-1,4-glucanase (EC 3.2.1.73); exo-β-1,4-glucanase/cellodextrinase (EC 3.2.1.74); cellobiohydrolase (EC 3.2.1.91); xyloglucan-specific endo-β-1,4-glucanase/endo-xyloglucanase (EC 3.2.1.151); exo-β-glucosaminidase (EC 3.2.1.165)
	GH48	Reducing end-acting cellobiohydrolase (EC 3.2.1.176); endo-β-1,4-glucanase (EC 3.2.1.4); chitinase (EC 3.2.1.14)
Hemicellulose	GH5 (subfamily 21)	Endo-β-1,4-xylanase (EC 3.2.1.8); xyloglucan-specific endo-β-1,4-glucanase (EC 3.2.1.151); arabinoxylan-specific endo-β-1,4-xylanase (EC 3.2.1.-)
	GH10	Endo-1,4-β-xylanase (EC 3.2.1.8); endo-1,3-β-xylanase (EC 3.2.1.32)
	GH11	Endo-β-1,4-xylanase (EC 3.2.1.8); endo-β-1,3-xylanase (EC 3.2.1.32)
	GH43	β-Xylosidase (EC 3.2.1.37); α-L-arabinofuranosidase (EC 3.2.1.55); arabinanase (EC 3.2.1.99); xylanase (EC 3.2.1.8); galactan 1,3-β-galactosidase (EC 3.2.1.145); α-1,2-L-arabinofuranosidase (EC 3.2.1.-); exo-α-1,5-L-arabinofuranosidase (EC 3.2.1.-); [inverting] exo-α-1,5-L-arabinanase (EC 3.2.1.-); β-1,3-xylosidase (EC 3.2.1.-)
	CE1	Acetyl xylan esterase (EC 3.1.1.72); cinnamoyl esterase (EC 3.1.1.-); feruloyl esterase (EC 3.1.1.73)
	CE6	Acetyl xylan esterase (EC 3.1.1.72)

#### Selection of CAZyme Families

Carbohydrate-active enzyme families were principally selected based on known mechanisms of enzymes and genomic data from fibrolytic microorganisms. Families GH5, GH9, and GH43 are present in large numbers in the genome of fibrolytic bacteria as well as families GH9 and GH48 in fungal genomes (Berg-Miller et al., 2009; Purushe et al., 2010; Suen et al., 2011; Pajon et al., 2013, Unpublished; Youssef et al., 2013). Additionally, cellulases from GH9 and GH48 are key enzymes of cellulosomes of *R. albus* and fungi (Devillard et al., 2004; Arai et al., 2006). These enzymes have potentially a significant role in the degradation of crystalline cellulose (Davison and Blaxter, 2005). Furthermore, endoglucanases from GH5 and GH9 have shown synergistic activity with GH48 cellobiohydrolases (Irwin et al., 2000). Regarding hemicellulose degradation, families GH5 (members of the subfamily 21), GH10, GH11, and GH43 are key enzymes of this process. Families GH10 and GH11 are almost exclusively constituted of endoxylanases that hydrolyse β(1,4) linkages of the xylan backbone. They have also been identified in xylanase utilization systems (XUS) of xylanolytic Bacteroidetes (Dodd et al., 2010). Endoxylanases from family GH5 are present in XUS systems suggesting a substantial role in xylan degradation (Aspeborg et al., 2012). Family GH43, retrieved in large number of copies in genomes of *Bacteroides* spp., is polyspecific. Although some xylanases are found in this family, it contains mainly xylosidase and debranching enzymes like arabinofuranosidases^4^ (Lombard et al., 2014). Finally, two families of CE (CE1 and CE6) were also selected. The family CE1, for which a high number of copies is retrieved from genomes of bacteria and fungi, is mainly composed of acetyl-, feruloyl-, and cinnamoyl-esterases, but other enzymes such as carboxylesterases that are not involved in fiber degradation, are also found in this family (Lombard et al., 2014)^4^. Feruloyl esterases, owing to their ability to dissociate lignin from hemicelluloses, have a pivotal role in xylan degradation by making polysaccharides accessible to other enzymes (Girio et al., 2010). The family CE6 is exclusively constituted of acetyl esterases that release acetyl groups from the xylan backbone. Their activity is also necessary to allow xylanolytic enzymes to access to their substrate (Faulds et al., 2006; Gottschalk et al., 2010; Zhang et al., 2011).

These eight families have been found among the most transcribed in the published rumen metatranscriptomes (Dai et al., 2015; Li and Guan, 2016; Shinkai et al., 2016; Comtet-Marre et al., 2017). Moreover, transcriptomic data obtained from *Prevotella bryantii* B_1_4 (Dodd et al., 2010), *Ruminococcus flavefaciens* FD-1 (Berg-Miller et al., 2009), *B. xylanisolvens* XB1A (Despres et al., 2016), and *Orpinomyces* sp. C1A (Youssef et al., 2013) have identified these families as up-regulated in pure culture with polysaccharide compared to simple sugar substrates. The targeted GH families were also among the 20 most expressed GH families detected by the CAZyChip, a DNA microarray targeting more than 55,000 bacterial GH genes, after 5 days of fermentation of WS by a simplified rumen microbial community (Abot et al., 2016). Finally, four out of the eight targeted CAZyme families (GH5, 43, 48, and CE1) on a total of 16 GH families identified with a metaproteomic approach were found in abundance in the rumen of cows fed corn silage, grass silage, or hay (Deusch et al., 2017). Hence, induction of genes encoding these families and their high expression in rumen metatranscriptomes suggests their strong contribution to the cellulose and hemicellulose degradation process.

#### Genes Targeted and Probe Design

In total, 392 nucleic sequences encoding 363 GH and 29 CE were kept for the microarray design. These sequences originated from 41 bacterial species, 4 protozoal species, and 10 fungal species (Supplementary Table S1). The main fibrolytic bacterial genera targeted were *Fibrobacter*, *Ruminococcus*, *Prevotella*, *Bacteroides*, *Butyrivibrio*, and *Cellulosilyticum*. Other gut bacterial genera known to degrade oligosaccharides and soluble polysaccharides, i.e., *Bifidobacterium*, were also targeted as well as some anaerobic fibrolytic bacteria from the genera *Clostridium* and *Cellulomonas* since they are likely present in the digestive ecosystems even if they are not essential to plant cell wall degradation (Li et al., 2013; Ziemer, 2014). Regarding commensal rumen eukaryotes, the fungal genera *Piromyces*, *Neocallimastix*, and *Orpinomyces*, and the protozoal genera *Epidinium*, *Eudiplodinium*, and *Polyplastron* were targeted (Supplementary Table S1). The microarray was composed of 1631 25-mer probes, ensuring the specific recognition of either unique sequences or groups of very closely related genes. Indeed, for sequences with more than 96% of identity, mostly originated from the same microbial species, consensus sequences were determined leading to 54 sets of 2–19 sequences for probe design. In summary, an average of five 25-mer probes per target gene was obtained (up to 14 probes per gene when a unique gene was targeted and up to 10 probes in case of a group of genes). Additionally, the GoArrays strategy, consisting in associating two probes targeting the same gene (Rimour et al., 2005), led to the determination of 2618 composite probes of 54-mers (GoArrays probes) with an average of 9 probes per target gene (up to 22 probes per unique gene and up to 37 probes in case of a group of genes). Finally, the FibroChip was constituted by a total of 4249 probes (Supplementary Table S2). All the probes targeted a gene region coding for the catalytic domain of the enzyme, which carries the activity (Supplementary Table S3). We did not target regions coding for accessory domains (CBM and dockerins), as these modules are sometimes associated with other proteins than CAZymes (Peer et al., 2009).

### Evaluation of the FibroChip Sensitivity and Specificity

The sensitivity of the probes defined by their ability to accurately recognize genes involved in the fiber degradation was assessed using seven genes amplified by PCR. These genes were selected in order to represent the diversity of targeted CAZyme families and microorganisms (**Table 2**), and were cloned to ensure that a unique sequence was hybridized. Around 50 ng of each gene, representing ∼10^10^ copies, was hybridized. Additionally, 1 μg of gDNA (∼10^11^ copies of genome) from three strains of fibrolytic bacteria (two rumen strains *F. succinogenes* S85 and *R. albus* 20, and one strain from the human gut *B. xylanisolvens* XB1A) was used (**Table 3**). The hybridization results show that all the probes gave a strong positive signal (**Tables 2**, **3**). GoArrays probes presented a better sensitivity than short probes, with a SNR of 5- to 17-fold higher for PCR products (not shown for gDNA), confirming the high sensitivity.

**Table 2:** Microarray results obtained by hybridization of seven cloned genes encoding different CAZymes from diverse microorganisms targeted by the FibroChip.

Target genes	25-mer probes			GoArrays probes			Other detected genes
	Positive probes^a^/total probes	Gene SNR^b^	Detection	Positive probes^a^/total probes	Gene SNR^b^	Detection	
GH5 *F. succinogenes* S85	5/5	312	+	9/9	3225	+	0
GH9 *R. albus* 20	6/6	474	+	8/8	2403	+	0
GH10 *B. xylanisolvens* XB1A	4/4	157	+	4/4	2166	+	0
GH11 *R. albus* 7	11/11	166	+	11/11	2860	+	0
GH48 *R. albus* 20	6/6	175	+	6/6	1939	+	0
GH10 *P. multivesiculatum*	6/6	152	+	8/8	2573	+	0
GH9 *Piromyces* sp. M4	6/6	272	+	14/14	2433	+	0

*^a^Number of probes that gave a positive signal (positive probes). ^b^SNR: signal of hybridization defined as a signal-to-noise ratio.*

**Table 3:** Microarray results obtained after hybridization of genomic DNA from selected bacterial strains.

Bacterial strain	Detected genes^a^/targeted genes^b^	Cross-hybridizations^c^	Other detected genes (origin)
*F. succinogenes* S85	31/31	2	1 (*Fibrobacter* sp.)
*B. xylanisolvens* XBlA	20/20	0	0
*R. albus* 20	2/2	44	3 (*R. albus* 8)
*E. coli* K12	0/0	0	0

*^a^Number of genes detected with the two probe types. ^b^Number of genes from the specific strain targeted by the FibroChip. ^c^Number of probes (25-mers and GoArrays) that led to unexpected hybridization.*

The genomes of rumen fungi harbor multicopies of GH48 genes of which nucleic sequences could reach 99% of identity (Youssef et al., 2013). This observation was confirmed for the strain *Piromyces* sp. M4 for which several GH48 genes were found (Forano et al., Unpublished data). Thereby, detection of sequence variants of genes used for probe design can be particularly useful (Dugat-Bony et al., 2012b). The capacity of FibroChip probes to detect these sequences was evaluated by hybridizing a GH48 clone from *Piromyces* sp. M4 (GenBank accession number MF773967), containing a sequence with 95% identity to the ones from other strains of *Piromyces* sp. used for probe design. The gene hybridized with 25-mer probes with perfect match, but no hybridization occurred with probes containing mismatches (Supplementary Table S6) confirming the high specificity expected for the short probes. GoArrays probes constituted by two perfectly matching probes and by one perfectly matching probe combined with one probe containing mismatches gave also a signal. The strongest signal was measured with the two perfectly matching probes (Supplementary Table S6). Besides, the GoArrays probes constituted by the two probes with mismatches did not produce any hybridization signal (not shown). This result demonstrates the exploratory potential of the FibroChip, which could be particularly useful to detect genes that are present in high number of variants such as GH48 and GH9 genes in rumen fungi (Youssef et al., 2013).

### Application of the FibroChip to Targeted Analysis of the Transcriptome of *Fibrobacter succinogenes* S85

Transcriptomic and proteomic data have shown that gut fibrolytic microorganisms are able to adapt the expression and production of CAZymes according to the substrate available (Béra-Maillet et al., 2000; Berg-Miller et al., 2009; Despres et al., 2016). To test the ability of the FibroChip to detect the differential expression of genes, the microarray was used to analyze the transcriptome of the rumen bacterium *F. succinogenes* S85 cultivated in two media differing by their carbon source, i.e., the simple sugar Clb or the complex substrate WS. Indeed, *F. succinogenes* S85 is known to degrade efficiently cellulose and xylans even if it cannot use xylose (Matulova et al., 2008). The substrate WS was essentially composed of cellulose, hemicelluloses (xylans), and lignin, and comprised 81% carbohydrates, with a monomer composition of 58% glucose and 34% xylose. WS was assumed to induce the expression of the *F. succinogenes* cellulase and hemicellulase genes targeted by the microarray. On both substrates, all the 31 genes targeted by the microarray were detected as expressed (GoArrays results, SNR threshold of 6 and at least 65% of probes positive). The SNR threshold value of 6 ensured a very specific response of the FibroChip.

**FIGURE 1:**
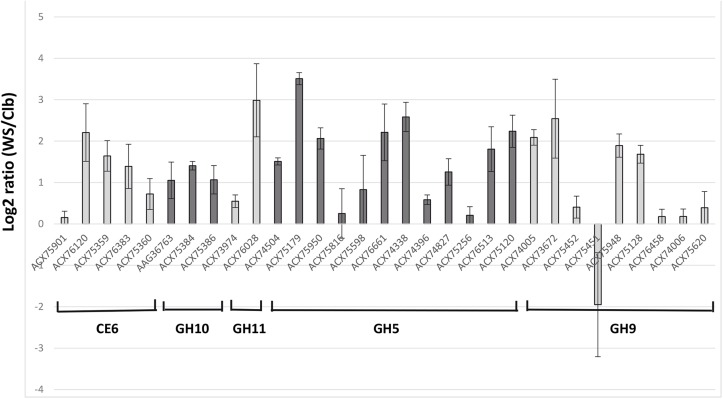
Microarray gene expression analysis of the targeted genes from *Fibrobacter succinogenes* S85 cultivated with cellobiose (Clb) or wheat straw (WS) as a sole substrate. Relative expression of expressed genes on WS versus Clb is expressed as log2 ratio (WS/Clb) ± SEM. Genes are denominated by the accession number of the protein encoded.

During growth in WS relative to Clb, nine genes were considered to be up-regulated based on the log2 ratio > 2 (**Figure 1**). They belonged to all the targeted CAZyme families, except GH10. The most induced genes (log2 ratio > 2.5) encoded a GH11 endo-β-1,4-xylanase (protein accession number ACX76028), the GH5 cellodextrinase CedA (ACX75179), a GH5 not yet characterized (ACX74338), and a GH9 endo-β-1,4-glucanase (EGB, Cel9G; ACX73672). The genes encoding EGB and CedA were also previously found as the most expressed genes by *F. succinogenes* S85 grown on cellulose *in vitro*, and were also among the most *F. succinogenes* expressed genes in the rumen of sheep (Béra-Maillet et al., 2000, 2009). The transcripts of a GH9 endoglucanase (EGE or CelE; ACX75451) were the only ones with a negative log2 ratio value. The expression of *celE* was then assessed by RT-qPCR. Expression of this gene was quantified at 1.4 × 10^5^ ± 2.8 × 10^3^ copies/ng of RNA on Clb and at 7.4 × 10^4^ ± 1.0 × 10^4^ copies/ng of RNA on WS, confirming the results of the DNA microarray. However, CelE (ACX75451) has been identified as a major cellulase EG1 produced by *F. succinogenes* S85 cultivated on cellulose, accounting for 32% of the endoglucanase activity released in the culture medium of the bacterium grown in a chemostat (McGavin and Forsberg, 1988; Malburg et al., 1996). Several hypotheses could be proposed to explain our results, such as the regulation of expression of this gene according to the growth stage of the bacteria, higher induction on Clb or WS structure, and composition resulting in more or less release of repressor compounds from WS. This gene should undergo complementary studies as well as other genes encoding enzymes not characterized yet, and which were over-expressed on WS substrate, such as the genes coding for GH5 ACX74338 and ACX76661. The sequencing of the genome of *F. succinogenes* S85 (Lucas et al., 2009, Unpublished) has identified a large number of GH (102) and CE (16) encoding genes^5^ (Lombard et al., 2014), but biochemical characterization of the proteins on different substrates remains necessary to confirm the enzymatic activities predicted by bioinformatics approaches. Targeted transcriptomics by DNA microarray may give opportunities to enhance our knowledge on how *F. succinogenes* S85 acts and identify which genes or proteins deserve further characterization even though the FibroChip microarray does not target all CAZyme genes present on its genome.

### Application of the FibroChip to a Targeted Metatranscriptomic Analysis of the Rumen Microbiota

**FIGURE 2:**
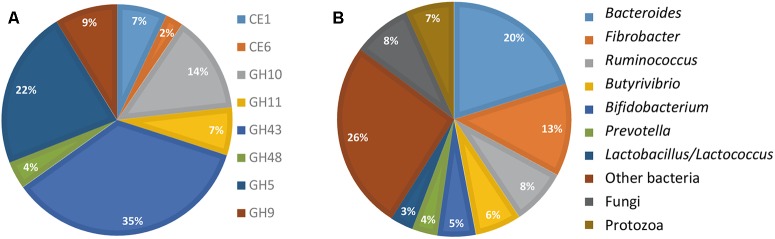
FibroChip results obtained after hybridization of RNA from rumen contents of a cow. **(A)** Relative contribution of the CAZyme transcripts detected by the FibroChip. **(B)** Taxonomic origin of the CAZyme transcripts detected by the FibroChip. Results presented are obtained with the GoArrays probes. A transcript was considered as detected when its SNR was >2 and at least 65% of the probes were positive. The relative SNR value was not taken into account for the graph representation. CAZyme families **(A)** and microorganism origin **(B)** were picked from CAZy (http://www.cazy.org) and taxonomic data of each detected transcript.

The FibroChip was then used to analyze the metatranscriptome of a cow rumen content. The rumen sample was from a dairy cow fed a mixed diet and was previously analyzed for fibrolytic activity by a RNA-seq metatranscriptomic approach (Comtet-Marre et al., 2017). For complex ecosystems, the SNR threshold is usually set at lower values than for pure cultures (Dugat-Bony et al., 2012a). Therefore, for rumen RNA hybridizations, the SNR threshold was set at 2 (He and Zhou, 2008). Only results from GoArrays were used because long probes were more adapted to complex samples. The results obtained by the GoArrays probes are given in **Figure 2**. The most numerous transcripts identified by the FibroChip coded for GH43, GH5, and GH10 enzymes (**Figure 2A**). *Bacteroides*, *Fibrobacter*, and *Ruminococcus* were the major contributors to the GH families targeted by the FibroChip, while fungi and protozoa represented 8.1 and 6.7% of total activity, respectively (**Figure 2B**). Among the 31 *F. succinogenes* S85 genes targeted by the FibroChip, 29 were detected by the GoArrays probes as expressed in the cow rumen. The transcripts not detected were those of a CE6 (ACX75901) and a GH9 (ACX75620) gene. Interestingly, transcripts from the gene *celE* (ACX75451), which was found down-regulated on WS compared to Clb in our *in vitro* experiment, were detected in the cow rumen, indicating that this gene can be expressed *in vivo* on complex substrates. The expression of four *F. succinogenes* S85 genes (coding for GH10 ACX75384, GH9 ACX73672 and ACX75948, and CE6 ACX75360) in the cow rumen was confirmed using RT-qPCR (not shown).

**FIGURE 3:**
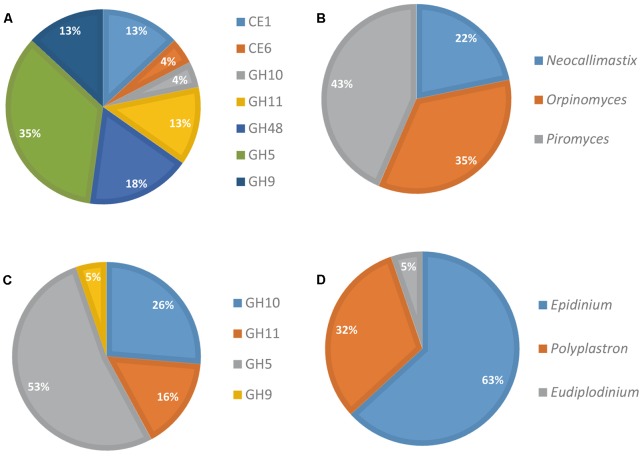
Distribution of fungi and protozoa to CAZyme transcripts in rumen contents of a cow. Relative contribution of fungi **(A)** and protozoa **(C)** CAZyme mRNA detected by the FibroChip; taxonomic origin of fungi **(B)** and protozoa **(D)** transcripts detected by the FibroChip. Results presented are obtained with the GoArrays probes. A transcript was considered as detected when its SNR was >2 and at least 65% of the probes were positive. The relative SNR value was not taken into account for the graph representation. CAZyme families **(A,C)** and microorganism origin **(B,D)** were picked from CAZy (http://www.cazy.org) and taxonomic data of each detected transcript.

The contribution of eukaryotes was also analyzed further (**Figure 3**). Fungi produced a diversified array of GH transcripts, covering all but one targeted GH families (**Figure 3A**), while protozoa produced mainly GH5 and to a lesser extent GH10 and GH11 transcripts (**Figure 3C**). The genus *Epidinium* appeared as a major contributor to the production of transcripts from the targeted CAZyme families. However, it should be noted that sequences from rumen fungi and protozoa are still very scarce in databases, and the FibroChip was designed according to the sequences known at the time of the design. The snapshot given by the FibroChip may be biased, particularly for the rumen eukaryotes.

In order to further assess the specificity of the GoArrays probes and confirm the FibroChip data for the rumen sample, transcripts of a *P. communis* GH11 gene (AAG18439) detected by the FibroChip were amplified by RT-PCR and isolated by cloning. Isolated sequence showed 92% identity with the targeted GH11 gene (AAG18439), suggesting that it was a variant of this gene. Such results illustrated the exploratory potential of the FibroChip microarray with a complex microbial community.

### Comparison of Microarray and RNA-Seq Data

**FIGURE 4:**
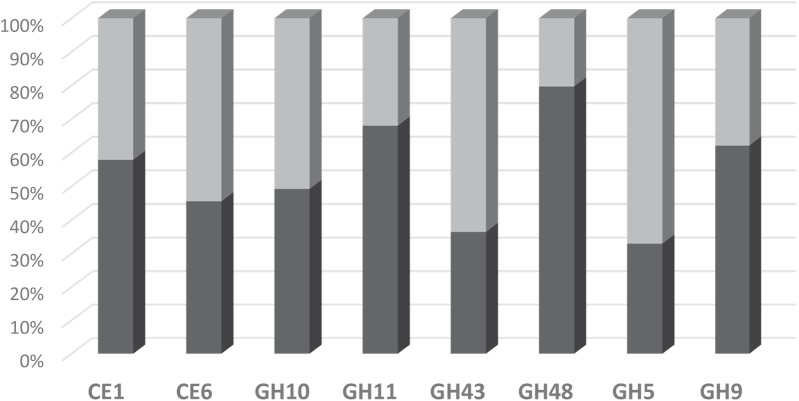
Distribution of detected transcripts into CE and GH families as assessed by the FibroChip (light gray) or the RNA-seq analysis (dark gray). Fifty percent indicates that the contribution of one family to the total of the targeted families is equally estimated by both methods.

The suitability of the FibroChip microarray was then evaluated against RNA-seq analysis performed on the same rumen sample (Comtet-Marre et al., 2017). The distribution of transcripts into the eight CAZyme families as assessed by the two high-throughput approaches was compared (**Figure 4**). The two methods gave quite similar results for transcripts assigned to families CE1, CE6, GH9, and GH10. However, a discrepancy was observed for the other four families GH11, GH43, GH48, and GH5, which were either under-estimated (GH11, GH48) or over-estimated (GH43, GH5) by the DNA microarray. In the RNA-seq dataset, there was a high proportion (15%) of GH11 sequences affiliated to “uncultured bovine ciliate from environmental samples,” which were probably not trapped by the FibroChip, possibly explaining the divergences between the two results. Concerning GH48, there was a clear under-estimation of bacterial GH48 transcripts by the microarray. Indeed, bacteria accounted for 89.4 and 36.4% of total GH48 transcripts in the RNA-seq and FibroChip data, respectively. These results indicated that the FibroChip must be completed by integrating new sets of probes targeting new sequences from GH11 and GH48 families which are now available in the databases. Regarding GH5 the differences between FibroChip and RNA-seq data were mainly due to sequences affiliated to fungal GH5. Indeed, the fungal GH5 transcripts represented 12.7 and 0.5% of the total GH5 sequences as measured by the FibroChip and RNA-seq analysis, respectively. For GH43, the differences between the two approaches were distributed on several taxons. Finally, the over-estimation of transcripts affiliated to families GH5 and GH43 by the FibroChip might result from the under-estimation of other families with the microarray (GH11 and GH48), which would thus lead to different proportions of CAZyme families. In addition, the methodologies used for RNA-seq annotation and taxonomic affiliation of CAZymes transcripts (described in Comtet-Marre et al., 2017) may also introduce some bias in the results and thereby in the comparison.

The FibroChip data highlighted the important, probably still under-estimated, role of eukaryotes in rumen fiber degradation which was in line with the RNA-seq metatranscriptomic analysis. The RNA-seq data indicated that rumen fungi sequences accounted for 6% of the sequences from families targeted by the FibroChip which is close to what was found with the microarray (8%). Qi et al. (2011) showed a higher discovery rate of fungal CAZymes in the rumen of the muskox by metatranscriptomics compared to previous metagenomic studies; however, in other studies, a substantial number of fungal transcripts was not always identified (Edwards et al., 2017). This may depend on how the rumen sample was prepared, in particular if it included the solid phase to which rumen fungi are mostly associated, but also on the procedure of mRNA isolation (Elekwachi et al., 2017). It should also be noted that the FibroChip enabled detection of gene transcripts, which may not totally reflect the actual cellulolytic and hemicellulolytic corresponding activities. This highlights the needs to use complementary methods, such as metabolomics or metaproteomics, to understand more in detail the microbiome activity of the rumen ecosystem (Deusch et al., 2017).

## Conclusion

The new FGA, FibroChip, represents a high-throughput tool that would enable monitoring the gene expression of key rumen prokaryotic and eukaryotic microorganisms involved in cellulolysis and hemicellulolysis. It would be applicable to study temporal variations in CAZyme gene expression profiles in the rumen according to diet composition, environmental changes, supplementation of feed additives, etc. This monitoring could be applied in nutritional experiments involving a large number of animals, and would allow to better understand the mechanisms of control or modulation of the fiber-degrading activity of rumen microbiota. With the constant increase in available information in the genomic, metagenomic, and specific databases such as CAZy, it will be possible to update and enrich the current version of the FibroChip with new target genes, in particular for rumen eukaryotes.

## Author Contributions

SC-M, EF, FC-D, PM, and PP conceived and designed the experiments. AB conceived and performed the animal experiment. SC-M and OB designed the chip. SC-M performed the molecular analyses. SC-M, FC-D, and EF performed the data analysis and wrote the manuscript. All authors have read and approved the final manuscript.

## Conflict of Interest Statement

FC-D is an employee of Lallemand SAS Company. SC-M was also employed by Lallemand Company during part of this work. Lallemand contributed to the funding of this work. The other authors declare that the research was conducted in the absence of any commercial or financial relationships that could be construed as a potential conflict of interest.

## Footnotes

1 http://www.cazy.org

2 http://g2im.u-clermont1.fr/hispod/index.php

3 http://g2im.u-clermont1.fr/serimour/goarrays.html

4 http://www.cazy.org

5 http://www.cazy.org
