# Supplementary material for: FibroChip, a Functional DNA Microarray to Monitor Cellulolytic and Hemicellulolytic Activities of Rumen Microbiota
Source: Front Microbiol. 2018 Feb 13;9:215. doi: 10.3389/fmicb.2018.00215 (PMC5816793; doi:10.3389/fmicb.2018.00215)
Supplement: Supplementary file 4 [file Data_Sheet_4.docx]

Supplementary Material

**FIBROCHIP, A FUNCTIONAL DNA MICROARRAY TO MONITOR CELLULOLYSIS AND HEMICELLULOLYSIS ACTIVITIES OF RUMEN MICROBIOTA**

**Sophie Comtet-Marre, Frédérique Chaucheyras-Durand, Ourdia Bouzid, Pascale Mosoni, Ali R. Bayat, Pierre Peyret and Evelyne Forano***

*** Correspondence:** Evelyne Forano : evelyne.forano@inra.fr

# Supplementary Tables

**Table S1.** Number of genes and corresponding CAZyme families targeted by the FibroChip for each species of microorganisms.

Excel file data sheet 1

**Table S2.** Summary of the designed probes. Type of design, probe length and identification are given according to the targeted CAZyme and microorganism.

Excel file data sheet 2

**Table S3.** List of the 392 CAZymes whose genes are targeted by the FibroChip microarray. For each CAZyme, name, accession number and family are indicated. The microorganism carrier of the gene is also specified.

Excel file data sheet 3

**Table S4.** Genes and plasmids used for the FibroChip validation.

| GH family | Protein name / accession number^a^ | Microorganism | Reference | PCR Primers | Temp  (°C)^b^ |
| --- | --- | --- | --- | --- | --- |
| GH5 | Cel5G / AAA24893 | *F. succinogenes* S85 | This work | F-CATAAAACCGACCCCAAAT  R-ATTGCGCCATTCCTGTTACT | 55 |
| GH11 | XynA / AAA85198 | *R. albus* 7 | This work | F-ATTTAGGTGACACTATAG  R-TAATACGACTCACTATAGGG | 40 |
| GH9 | Cel9 / MF773966 | *Piromyces sp.* M4 | This work | F-GAAGCTGGTCAATGGG  R-GGAGCGTGCCATCCGTC | 45 |
| GH48 | Cel48 / MF773967 | *Piromyces sp.*  M4 | This work | F-CTCTTGCTCTTCTCGGTGCT  R-GGTGGGTTACCTCCGTTAAG | 51 |
| GH9 | Cel9B / CAS03458 | *R. albus* 20 | Rakotoarivonina et al. (2009) | F-CAAAAATCTAGATAACGAGGGC  R-CTGAGACCATGGTCCCC | 55 |
| GH10 | Xyn10A / CBH32823 | *B. xylanisolvens* XB1A | Mirande et al. (2010) | F-CCCGAATTCGAGCTCGG  R-GATGGTGATGCGATCCTC | 56 |
| GH48 | Cel48A / CAS03459 | *R. albus* 20 | Rakotoarivonina et al. (2009) | F-CCCGAATTCGAGCTCGG  R-GATGGTGATGCGATCCTC | 56 |
| GH10 | XynB / CAB65753 | *P. multi-vesiculatum* | Devillard et al. (2003) | F-ATGAAAAAGTCTACTATAGCATTCTTATTAATC  R-CGTTGTCAACAATAGAATAAAAGC | 55 |

^a^ Accession numbers were from Genbank

^b^ Temp: temperature used for primer hybridization in the PCR reaction

References

Devillard, E., Béra-Maillet, C., Flint, H.J., Scott, K.P., Newbold, C.J., Wallace, R.J., et al. (2003). Characterization of XYN10B, a modular xylanase from the ruminal protozoan *Polyplastron multivesiculatum*, with a family 22 carbohydrate-binding module that binds to cellulose. *Biochem. J.* 373, 495–503.

Mirande, C., Mosoni, P., Béra-Maillet, C., Bernalier-Donadille, A., and Forano, E. (2010). Characterization of Xyn10A, a highly active xylanase from the human gut bacterium *Bacteroides xylanisolvens* XB1A. *Appl. Microbiol. Biotechnol.* 87, 2097–2105.

Rakotoarivonina, H., Terrie, C., Chambon, C., Forano, E., and Mosoni, P. (2009). Proteomic identification of CBM37-containing cellulases produced by the rumen cellulolytic bacterium *Ruminococcus albus* 20 and their putative involvement in bacterial adhesion to cellulose. *Arch. Microbiol.* 191, 379–388.

**Table S5**. Primers used for the reverse transcription and the PCR amplification of a sequence variant of the gene encoding GH11 *Piromyces communis* (AAG18439).

|  | Primer | Sequence 5’-3’ |
| --- | --- | --- |
| Reverse transcription |  |  |
|  | AAG18439 192-216R | GGGTTTGAGTACTATCGAAG |
|  | AAG18439 221-245R | GCATATATGTGACCAATTT |
|  | AAG18439 347-371R | GGACGGTATTGACTGAGCC |
|  | AAG18439 429-453R | TTACGAAAACACTCGTTACGGTCCA |
| Nested PCR |  |  |
| Primary PCR |  |  |
|  | AAG18439 13-37F | AAGAACGCCTCTCACAGTGG |
|  | AAG18439 429-453R | TTACGAAAACACTCGTTACGGTCCA |
| Secondary PCR |  |  |
|  | AAG18439 13-37F | AAGAACGCCTCTCACAGTGG |
|  | AAG18439 221-245R | GCATATATGTGACCAATTT |

**Table S6.** SNR of the probes targeting the gene encoding a GH48 enzyme from *Piromyces* sp. M4 after the hybridization of a GH48 clone from the same strain containing several mismatches with the sequence used for the probe design.

|  |  | 25-mer probes | |  |  | GoArrays probes | | |
| --- | --- | --- | --- | --- | --- | --- | --- | --- |
|  | Probe | Probe sequence^a^ | SNR^b^ | Gene SNR |  | 25-mer probe association | SNR^b^ | Gene SNR |
| GH48 *Piromyces* sp. M4  (Genbank MF773967) | 1 | TC**TT**TAC**G**AC**G**TTATGACTGGTAA**C** | 1.4 (-) | **21.3** |  | 1_2 | 62.7 (+) | **2616.2** |
|  | 2 | TTAACACTTTCCAACGTGGTGCTTC | 43.3 (+) |  |  | 2_3 | 5141.2 (+) |  |
|  | 3 | TACTTCCATGGACCGTCAATTAGAA | 21.2 (+) |  |  | 2_4 | 3061.9 (+) |  |
|  | 4 | TACG**C**T**C**AACCAGA**T**AGTGACGTTA | 1.2 (-) |  |  | 3_4 | 137.7 (+) |  |
|  | 5 | AAACTACTCGTGTTAACGGTGGTCA | 39.4 (+) |  |  | 3_5 | 4677.3 (+) |  |

^a^ Differences in the nucleic sequence between the probes and the GH48 clone are highlighted in bold and underlined

^b^ Positive probes (with a SNR higher than the fixed threshold) and negative probes are indicated by a (+) and a (-) symbol, respectively
